# Physiological levels of adrenaline fail to stop pancreatic beta cell activity at unphysiologically high glucose levels

**DOI:** 10.3389/fendo.2022.1013697

**Published:** 2022-10-25

**Authors:** Nastja Sluga, Lidija Križančić Bombek, Jasmina Kerčmar, Srdjan Sarikas, Sandra Postić, Johannes Pfabe, Maša Skelin Klemen, Dean Korošak, Andraž Stožer, Marjan Slak Rupnik

**Affiliations:** ^1^ Faculty of Medicine, Institute of Physiology, University of Maribor, Maribor, Slovenia; ^2^ Center for Physiology and Pharmacology, Medical University of Vienna, Vienna, Austria; ^3^ Alma Mater Europaea, European Center Maribor, Maribor, Slovenia

**Keywords:** adrenaline, islets, beta cells, cAMP, concentration dependency, [Ca^2+^]_c_ oscillations, forskolin

## Abstract

Adrenaline inhibits insulin secretion from pancreatic beta cells to allow an organism to cover immediate energy needs by unlocking internal nutrient reserves. The stimulation of α2-adrenergic receptors on the plasma membrane of beta cells reduces their excitability and insulin secretion mostly through diminished cAMP production and downstream desensitization of late step(s) of exocytotic machinery to cytosolic Ca^2+^ concentration ([Ca^2+^]_c_). In most studies unphysiologically high adrenaline concentrations have been used to evaluate the role of adrenergic stimulation in pancreatic endocrine cells. Here we report the effect of physiological adrenaline levels on [Ca^2+^]_c_ dynamics in beta cell collectives in mice pancreatic tissue slice preparation. We used confocal microscopy with a high spatial and temporal resolution to evaluate glucose-stimulated [Ca^2+^]_c_ events and their sensitivity to adrenaline. We investigated glucose concentrations from 8-20 mM to assess the concentration of adrenaline that completely abolishes [Ca^2+^]_c_ events. We show that 8 mM glucose stimulation of beta cell collectives is readily inhibited by the concentration of adrenaline available under physiological conditions, and that sequent stimulation with 12 mM glucose or forskolin in high nM range overrides this inhibition. Accordingly, 12 mM glucose stimulation required at least an order of magnitude higher adrenaline concentration above the physiological level to inhibit the activity. To conclude, higher glucose concentrations stimulate beta cell activity in a non-linear manner and beyond levels that could be inhibited with physiologically available plasma adrenaline concentration.

## Introduction

Pancreatic endocrine cells have a prominent role in maintaining plasma nutrient levels. In physiological hyperglycemia, beta cells secrete insulin to move excess glucose into target cells and to eventually terminate their own activation. During the episodes of acutely increased glucose consumption during stress, beta cells must be switched off. In contrast, alpha cells respond with an increased glucagon secretion to support the activity of several other hormonal systems in an organism to recover and maintain the adequate plasma glucose level. The release of hormones from both beta and alpha cells is regulated by the autonomic nervous system (1–3).

The release of adrenaline from chromaffin cells of the adrenal medulla results in elevated blood glucose level, that follows glucagon secretion and stimulation of gluconeogenesis and glycogenolysis (4, 5), and inhibition of insulin release from beta cells (2, 6). Many factors, besides cellular reuptake, short half-time due to kidney clearance, enzymatic degradation, environmental stress, and food intake (7) affect the plasma concentration of catecholamines, making a reliable plasma catecholamine level hard to assess accurately (8). Moreover, catecholamines are light sensitive and can be oxidated to adrenochrome with different pharmacological properties. The physiological resting plasma adrenaline and NA concentrations are therefore low, in 10^-10^ M range (5). During exercise in humans, these levels can increase to some nM range (9). Different euthanasia methods contribute to the variability in the measurement of plasma catecholamines levels. The latest analytical methods and techniques for collecting animal blood without animal handling, and further measures to reduce environmental stress, suggest that previously measured plasma catecholamines levels have typically overestimated the plasma adrenaline concentration (10, 11). In C56BL/6J mice, resting plasma adrenaline levels and levels after euthanasia with CO_2_, followed by decapitation have been estimated to be in the range of 0.5-10 nM (12). As a result, so far only a handful of studies have addressed adrenergic effects on pancreatic beta cell matching these physiological conditions (13–15). Without a doubt, a commonly used higher adrenaline concentrations have been successfully used to discriminate between beta and alpha cells in different pancreas preparations (16, 17).

The effect that adrenaline has on a target tissue depends on distribution of adrenergic receptor types. In pancreas, adrenaline mediates their effects in pancreas by acting on α - and β-adrenergic receptors (4). All of them are members of G-protein coupled receptors family (GPCR). In endocrine pancreas all three subtypes of α_2_-adrenoceptors and β_2_-adrenoceptors have been discovered (18, 19). Recent studies indicate that a subtype α_2_A-adrenoceptor is the most abundant in mouse (20) and human pancreatic islets (4). α2-receptors mediate the cellular signaling through the G_i_-dependent mechanism, leading to inhibition of adenylyl cyclase (AC) activity. Actions mediated through β-adrenergic receptors are G_s_ mediated, and therefore the binding of agonists to β-adrenergic receptors stimulates AC activity (5).

The published experimental evidence associates the inhibition of insulin secretion with activation of α_2_A- or α_2_C adrenoceptors (4, 13, 21). Yohimbine, an α_2_A-adrenoceptor antagonist selectively prevents inhibition by adrenaline (13, 22). Along the same lines, α_2_A-adrenoceptor inhibitory effect on insulin secretion has been further confirmed by using α_2_A-adrenoceptor knockout (α_2_-KO) mice (20). Recently, a correlation between the reduced ability of beta cells to secrete insulin and polymorphism in the human α_2_A-adrenoceptor gene (*ADRA2A*) has been established (23). On the other hand, an increased α_2_A-adrenergic receptor expression has been associated with reduced insulin secretion and increased risk for developing type 2 diabetes (23).

The direct agonist binding to α_2_-adrenergic receptors on pancreatic beta cells (4, 13), has been originally described to influence several different cellular processes, like opening probability of ion channels, membrane potential, [Ca^2+^] homeostasis, and late steps of the regulated exocytosis of insulin (2, 13, 24). The common pathway following activation of α_2_-adrenergic receptors in beta cells is inhibition of AC and reduced production of cAMP. The concentration of cAMP in beta cells would typically be elevated after the GLP-1 binding to its receptor and activation of G_s_ mediated processes. cAMP in beta cells has been found to work through either protein kinase A (PKA)- or guanine nucleotide exchange factor 2 (Epac2A)-dependent pathways (25).

The open question is to what extent can glucose directly influence the production of cytosolic cAMP concentration in beta cells? More than half a century ago, it has been first suggested that glucose-induced insulin release is independent of cAMP production (26, 27). Soon after, evidence started to emerge suggesting that cAMP plays a prominent role in modulating glucose-induced release of insulin (28). It has been proposed that glucose, in addition to its function as a metabolic fuel, serves as a ligand for plasma membrane receptor (29, 30). Since then, experimental evidence preferentially supported the glucose fuel and cAMP modulatory concept (31, 32). Forskolin at increasing concentrations induced cAMP raise in the concentration range spanning at least two orders of magnitude to promote electrical activity, and raise [Ca^2+^]_c_, from both intracellular and extracellular sources (33). GLP1R signaling in β-cells has been reported to contribute to basal and glucose-induced cAMP production and insulin secretion (34). In addition, clinical work has shown that α_2_A-adrenoceptor antagonists, in clinical use as antipsychotics and antidepressants, potentiate the insulinotropic effect of drugs used in diabetes therapy, leading to severe adverse effects (4), supporting the modulatory role of cAMP on Ca^2+^-dependent insulin release in beta cells. Furthermore, glucose has been shown to drive submembrane fluctuations of cAMP (28, 35, 36), and sensitize the exocytosis machinery to Ca^2+^, thus promoting insulin exocytosis at lower Ca^2+^ concentration (35, 37). On the other hand, the glucose as receptor ligand concept developed mostly independently, uncovering the molecular complexity of glucose sensing with sweet taste receptors expressed in pancreatic beta cell (38, 39), however with relatively little intersection to the leading concept described above.

Adrenaline, on the other hand stimulates glucagon secretion *via* α_1_- and β-adrenergic receptors (40). The underlying mechanism has been demonstrated to involve activation of β-adrenergic receptors that is subsequently leading to increased levels of cAMP (5). cAMP presumably enhances glucagon secretion by mobilizing Ca^2+^ from intracellular stores, enhancing Ca^2+^ influx through plasma membrane and mobilizing secretory granules containing glucagon (40, 41). Additionally, to changes in [cAMP]_c_ activation of α_1_-adrenergic receptors on alpha cells results in increased levels of [Ca^2+^]_c_ (40).

In a majority of functional islets studies, unphysiologically high or low glucose concentrations have been used to assess functional properties of alpha and beta cells. High levels of adrenaline have been utilized to further differentiate between these two cell types in isolated islet and pancreatic slices. In this paper we demonstrate that stimulating beta cell collectives with physiological glucose concentration triggers [Ca^2+^]_c_ events, are readily inhibited by physiological adrenaline concentration levels. To inhibit beta cell stimulated with supraphysiological glucose levels requires progressively higher adrenalin concentration, which can be orders of magnitude above the physiological levels.

## Materials and methods

### Ethics statement

The study has been conducted strictly following all national and European (Directive 2010/63 EU) recommendations on the care and handling of experimental animals. All efforts to minimize the suffering of animals and to implement improvements in animal care and welfare were made. Administration of the Republic of Slovenia for Food Safety, Veterinary Sector and Plant Protection approved the experimental protocol (Licence No: U34401-12/2015/3) and so did The Ministry of Education, Science and Research, Republic of Austria (Licence No: 2020-0.488.800).

### Tissue slice preparation and dye loading

C57BL/6J mice of either sex, 10-45 weeks of age, were kept on a 12:12h light:dark (light 7 a.m.-7 p.m.) schedule in individually ventilated cages (Allentown) in standard conditions. Mice were used for pancreas tissue slices preparation as described before (42, 43). Briefly, mice were euthanized with CO_2_, and killed by cervical dislocation. Laparotomy was performed to access abdominal cavity. Next, we distally clamped the common bile duct at the major duodenal papilla, allowing low-melting point 1.9% agarose at 40°C (Lonza, Basel, Switzerland) to perfuse pancreatic ducts. Agarose was dissolved in extracellular solution consisting of (in mM) 125 NaCl, 2.5 KCl, 26 NaHCO_3_, 1.25 NaH_2_PO_4_, 2 Na pyruvate, 0.25 ascorbic acid, 3 myo-inositol, 6 glucose, 1 MgCl_2_, 2 CaCl_2_ and 6 lactic acid (ECS). Promptly after agarose injection, the perfused pancreas was cooled with ice-cold ECS, extracted, embedded into agarose blocks, and finally cut into 140 µM thick pancreas tissue slices using vibratome (VT 1000 S, Leica Microsystems). The successful injection of agarose is a critical step in pancreas slice preparation. Slices were collected in HEPES buffered solution (HBS) at room temperature composed of (in mM) 150 NaCl, 10 HEPES, 5 KCl, 2 CaCl_2_, 1 MgCl_2_; titrated to pH=7.4 using 1 M NaOH. For staining, slices were incubated for 50 min at room temperature in the dye-loading solution consisting of 6 µM Calbryte 520 AM, a Ca^2+^ sensitive indicator (AAT Bioquest), 0.03% Pluronic F-127 (w/v) and 0.12% dimethylsulphoxide (w/v) dissolved in HBS. If not specified otherwise, all chemicals were obtained from Sigma-Aldrich, St. Louis, MO, USA.

### [Ca^2+^]_c_ imaging and stimulation protocol

Imaging was performed on a Leica TCS SP5 upright confocal system using a Leica HCX APO L water immersion objective (20x, NA 1.0) or Leica TCS SP5 DMI6000 CS inverted confocal system using HC PL APO water/oil immersion objective (20x, NA 0.7). Acquisition frequency was set to 20 Hz at 256 x 256 pixels, pixel size to around 1 µm^2^. Calbryte 520 was excited by a 488 nm argon laser. Emitted fluorescence was detected and measured by a Leica HyD hybrid detector in the range of 500-700 nm with the standard or photon-counting mode (Leica Microsystems, Germany).

Before [Ca^2+^]_c_ imaging, pancreas tissue slices were kept at substimulatory glucose concentration (6 mM) in HBS. To avoid bias related to slices originating from different anatomic regions of pancreas, slices have been mixed and randomly picked up for imaging. After the preincubation period slices were transferred into an imaging perfusion system with 6 mM glucose in ECS and maintained at 37°C, after which ECS with physiological stimulatory (8 or 9 mM) or supraphysiological glucose concentrations (12, 16 or 20 mM) were used to stimulate [Ca^2+^]_c_ events. Adrenalin concentrations in the concentration range between 0.1 and 5000 nM have been used. Forskolin has been used at 100 and 500 nM concentrations.

### Analyses

The analysis of [Ca^2+^]_c_ events has been performed as previously described (44). Briefly, the movies were processed using custom Python script to automatically detect ROIs corresponding to individual cells. Within the detected ROIs the events were characterized by the start time, peakpoint time at the maximal amplitude, and the width of the pulse at the half of the height, which is a parameter we used to evaluate the duration of the event. The processing step included motion and phase correction. Beta cells were identified based on their typical activity pattern, being inactive at non-stimulatory glucose concentration (6 mM) and reacting in a biphasic manner when stimulated by 8 mM glucose. All cells outside histologically identifiable islet have been discarded from further analysis. In the next step, [Ca^2+^]_c_ events were automatically distilled and annotated from each ROI.

For this study, altogether 35 slices have been imaged. Out of these, 10, 8, 8, 9, and 1 slices were subjected to 8, 9, 12, 16, and 20 mM glucose, respectively. The time period of slice exposure to stimulatory glucose concentration was adjusted to let the beta cells to achieve a stable plateau activity (32, 44). Such a stable plateau activity was a prerequisite for sequent exposure to progressively higher adrenaline concentration to fully inhibit [Ca^2+^]_c_ events at all dominant time scales. To statistically compare the data we log transformed them. The mean value and its standard error has been computed from the mean values from individual islets. A one-way Anova and Bonferroni *post hoc* correction for multiple tests have been used to evaluate differences between pairs of treatment at significance level below 0.001.

## Results

**Figure 1:**
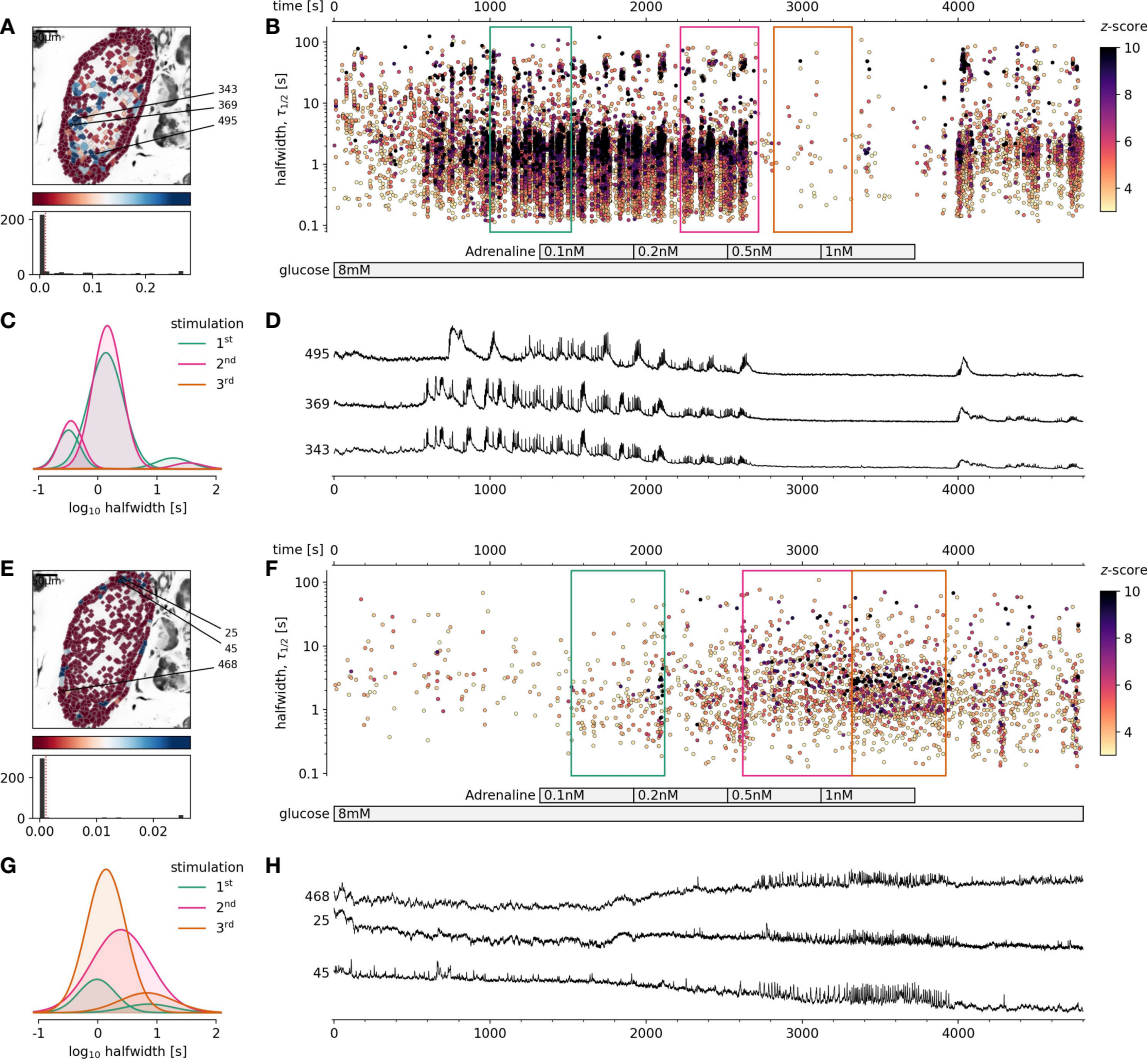
The effect of physiological adrenaline on beta and alpha cells activity at 8 mM glucose. **(A-D)** Beta cells, **(E-H)** Alpha cells. **(A, E)**, Regions of interest (ROIs) obtained by our segmentation algorithm. The color indicates the number of events identified in the ROI trace, upon a high-pass filtering at 0.2Hz. We discarded ROIs with number of events below the threshold (red dashed line in the histogram in the lower panel). Indicated are the ROI numbers whose filtered traces correlate best with the average trace for the whole islet. **(B, F)**, Events’ halfwidth duration through time. Note the ranges of halfwidth duration occurring, the events’ synchronicity, and a concentration-dependent block of the activity in beta cells, as well as activation in alpha cells with increasing adrenaline concentration. Color indicates the statistical significance in terms of z -score as indicated. The treatment protocol is indicated in the bar at the bottom of the pane. Due to a significantly larger number of ROIs corresponding to beta cells in comparison to alpha cells, this representation should not be used to compare the activity of both cell types directly. **(C, G)**, Normalized Gaussian fits through the logarithmic distribution of halfwidth duration, indicated temporal summation producing 3 discrete modes in beta cells and mostly 1 mode in alpha cells. **(D, H)**, Time courses from ROIs indicated in A and E, exposed to an increasing concentration of adrenaline, and rebinned to 2 Hz (recorded at 20 Hz).

This study was designed to evaluate the physiological effect of adrenaline on glucose stimulated [Ca^2+^]_c_ events in beta cell collectives. For this, confocal microscopy with high spatial and temporal resolution has been used to record changes in [Ca^2+^]_c_ over prolonged periods of time in pancreatic islet in situ. For the analysis we used custom-made Phyton scripts to first identify individual ROI, and second distill and annotate [Ca^2+^]_c_ events within these ROIs. [Ca^2+^]_c_ events were evoked by stimulatory glucose in the concentration range from 8 to 20 mM. Using a wide range of adrenaline concentrations, we attempted to completely suppress the activity elicited by glucose. As reported before, physiological glucose stimulation triggers [Ca^2+^]_c_ events in three primary time domains, ultra-short, dominant short and long events, reflecting different levels of temporal summation of ultra-short events (**Figures 1A–D**). At progressively higher glucose stimulation the densities redistribute in favor of longer events due to more temporal summation. A biphasic [Ca^2+^]_c_ response to glucose stimulation was composed of initial and mostly non-correlated transient phase with predominantly long events of mean duration of tens of seconds, followed by the cross-corelated plateau phase where short events with duration 2-3 s represented a dominant time domain.

### Adrenaline in physiological concentration inhibited glucose-dependent beta cell activity and stimulated alpha cell activity

In this study we demonstrated for the first time that using fresh pancreas tissue slices, it is possible to study the effects of physiological levels of adrenaline on [Ca^2+^]_c_ dynamics in alpha and beta cells exposed to physiological glucose levels. We showed that adrenaline in a concentration-dependent manner and in physiological concentration range differentially affected both pancreatic endocrine cell types (**Figure 1**). We found that adrenaline concentration that has been sufficient to inhibit glucose-dependent [Ca^2+^]_c_ events in beta cells, was sufficient to trigger [Ca^2+^]_c_ events in alpha cells in a concentration-dependent manner despite high glucose (**Figures 1E–H**). This enabled us to readdress the physiological role of sympathoadrenal system in islet cells in situ. In some islets stimulated with 8 mM glucose, even the lowest measured physiological concentration of adrenaline (0.5 nM), equivalent to non-stressed conditions was able to inhibit [Ca^2+^]_c_ events in some beta cell collectives. The effect of adrenaline was reversible and after a washout, cells returned to their normal activity. To summarize, when beta cells were stimulated with physiological glucose levels, physiological concentration of adrenaline in a low nanomolar range was sufficient to completely inhibit beta cell collective activity and to stimulate the alpha cell activity.

### Inhibitory effect of adrenaline on beta cells is glucose-dependent

**Figure 2:**
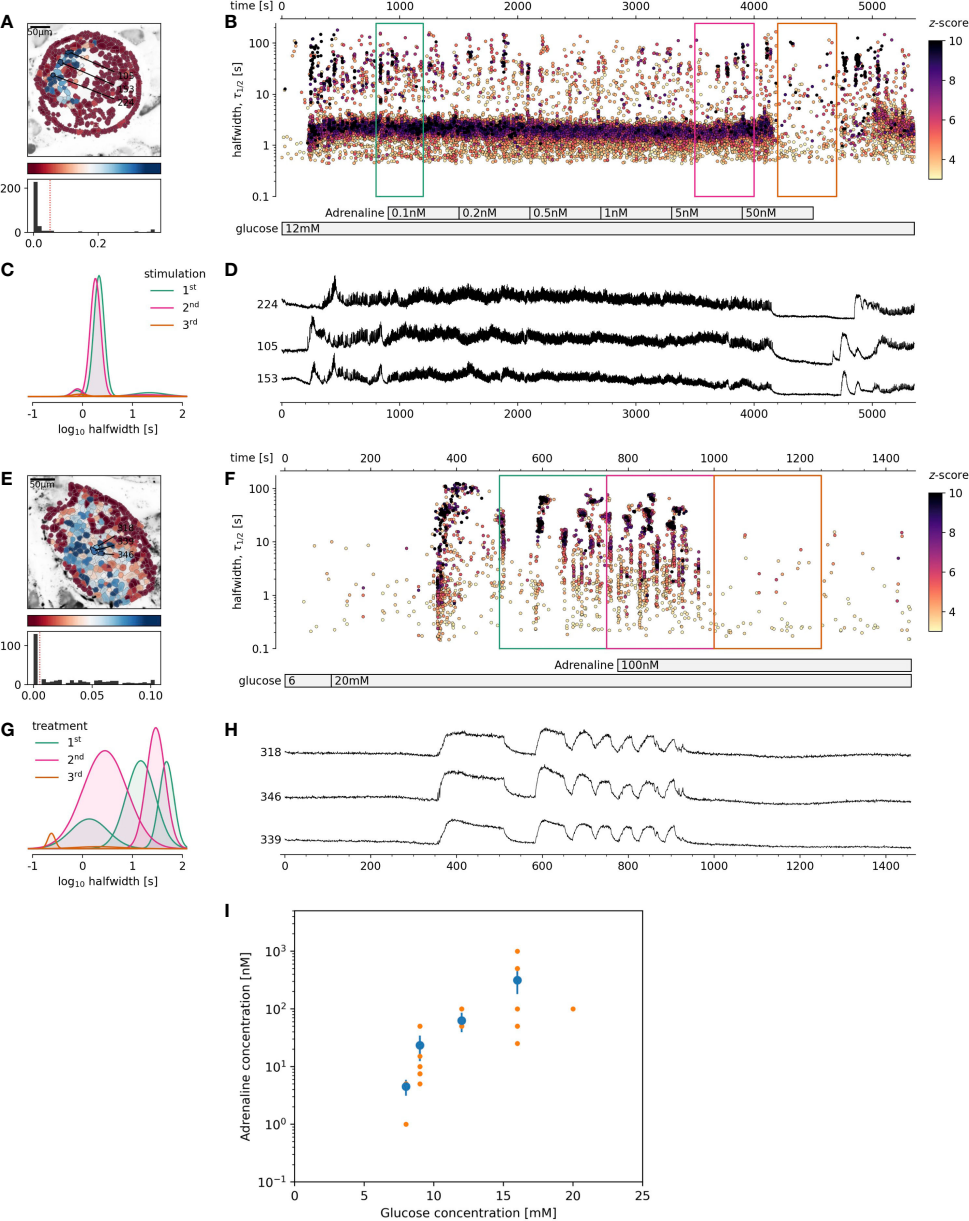
The effect of adrenaline on beta cell activity at supraphysiological glucose stimulation. **(A-D)** 12 mM glucose stimulation, **(E-H)** 20 mM glucose stimulation. **(A, E)**, Regions of interest (ROIs) obtained by our segmentation algorithm. The color indicates the number of events identified in the ROI trace, upon a high-pass filtering at 0.2Hz. We discarded ROIs with number of events below the threshold (red dashed line in the histogram in the lower panel). Indicated are the ROI numbers whose filtered traces correlate best with the average trace for the whole islet. **(B, F)**, Events’ halfwidth duration through time. Note the ranges of halfwidth duration occurring, the events’ synchronicity, and a concentration-dependent increase in frequency and halfwidth duration of [Ca^2+^]_c_ events. Color indicates the statistical significance in terms of z -score as indicated. The treatment protocol is indicated in the bar at the bottom of the pane. **(C, G)**, Normalized Gaussian fit through the logarithmic distribution of halfwidth duration, indicated temporal summation producing 3 discrete modes in beta cells. **(D, H)**, Time courses from ROIs indicated in **(A, E)**, exposed to an increasing concentration of adrenaline, and rebinned to 2Hz (recorded at 20Hz). **(I)**, Adrenaline concentration-dependent inhibition of glucose-dependent activation of beta cell collectives. The mean of islet means +/- SEM (blue dots and lines), and individual islet means (orange dots). The number of islets used for the plot was 10, 8, 8, 9, 1 for 8, 9, 12, 16 and 20 mM glucose, respectively. One-way Anova, followed by a Bonferroni correction for multiple tests revealed the differences at the level below 0.001 between 8 mM glucose and both 12 and 16 mM glucose.

The first question that arose from the high sensitivity of beta cells to adrenaline under physiological conditions was whether this sensitivity is conserved also with increased glucose load. Could low nM range of adrenaline inhibit activation of beta cell collectives stimulated with 12 mM or higher glucose? Exposure of the islets to progressively higher glucose concentration demanded a progressively higher adrenaline concentration to complete its inhibition (**Figure 2**). Despite the relatively high variability in inhibitory adrenaline concentration required for inhibition of the dominant time scale events at progressively higher glucose concentration, there was an evident and rather steep concentration-dependence, and progressively higher adrenaline concentrations were needed to stop the activity of beta cell collectives (**Figure 2**). It is therefore not surprising that isolated beta cells or islets, routinely stimulated with supraphysiological glucose concentration in a range between 15 and 25 mM, were found to have a rather low sensitivity to pharmacological inhibition with adrenaline. High concentration of adrenaline (5 µM) were needed to differentiate between pancreatic alpha and beta cells (16, 17).

**Figure 3:**
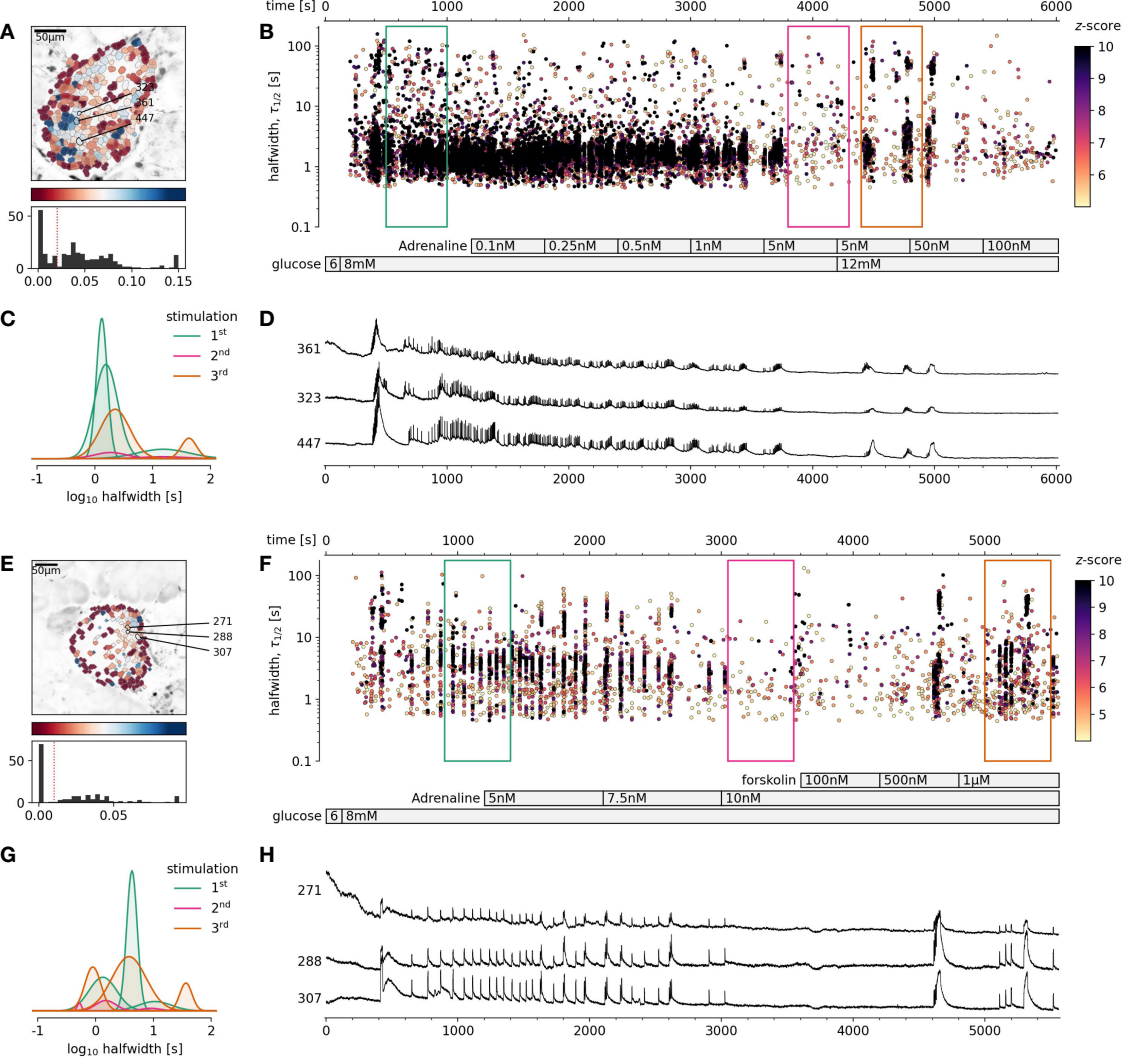
The rescue of adrenaline inhibition of 8 mM stimulated beta cell collective activity with higher glucose concentration or forskolin. **(A-D)** Rescue with 12 mM glucose, **(E-H)** Rescue with 500 nM foskolin. **(A, E)**, Regions of interest (ROIs) obtained by our segmentation algorithm. The color indicates the number of events identified in the ROI trace, upon a high-pass filtering at 0.2Hz. We discarded ROIs with number of events below the threshold (red dashed line in the histogram in the lower panel). Indicated are the ROI numbers whose filtered traces correlate best with the average trace for the whole islet. **(B, F)**, [Ca^2+^]_c_ events’ halfwidth duration through time. Color indicates the statistical significance in terms of z -score as indicated. The treatment protocol is indicated in the bar at the bottom of the pane. **(C, G)**, Normalized Gaussian fit through the logarithmic distribution of halfwidth duration. **(D, H)**, Time courses from ROIs indicated in **(A, E)**, exposed to an increasing concentration of adrenaline or forskolin, and rebinned to 2Hz (recorded at 20Hz).

Based on these results, the next question was, if stronger glucose stimulation could reverse adrenaline inhibition? A glucose increase to 12 mM, rather than 9 mM, was sufficient to override the inhibitory effect of adrenaline on beta cells (**Figures 3A–D**). This reactivation could in turn be inhibited again with an order of magnitude higher adrenaline concentration in comparison to that used to inhibit 8 mM glucose. Following the same pattern, the effect of adrenaline concentration, sufficient to inhibit beta cell activity at 12 mM glucose could be rescued by 16 mM glucose. Another order of magnitude higher adrenaline concentration has been required to inhibit beta cells activated at 12 mM glucose (**Figures 3A–D**).

### Direct pharmacological stimulation of cAMP production with forskolin restores beta cell activity after adrenaline-induced inhibition

Our next question has been whether high glucose concentration rescue of adrenaline inhibition of beta cell collectives could be reproduced by directly pharmacological targeting of the cAMP production? To assess this, we first stimulated islets with physiological levels of glucose, inhibited the response with adrenaline, and finally added forskolin, a direct stimulator of the adenylate cyclase, which has been previously described to raise the cytosolic cAMP concentration over several orders of magnitude in a concentration-dependent manner (33, 45). To fairly mimic the effect observed with glucose, we had to lower the concentration of forskolin from typically used 10 µM, to the nM range. Already 500 nM concentration of forskolin namely sufficed to reproduce the effect of higher glucose concentration (**Figures 3E–H**). At 10 µM forskolin, only very long [Ca^2+^]_c_ events, typically observed at very high glucose stimulation have been observed. To summarize, since forskolin alone was able to rescue [Ca^2+^]_c_ events, we suggest that in addition to raising [Ca^2+^]_c_ in beta cells, different levels of glucose, could similarly to forskolin, increase cytosolic cAMP concentration over several orders of magnitude.

### Sufficient level of cAMP does not trigger but is required to support coherent activation of beta cell collectives

**Figure 4:**
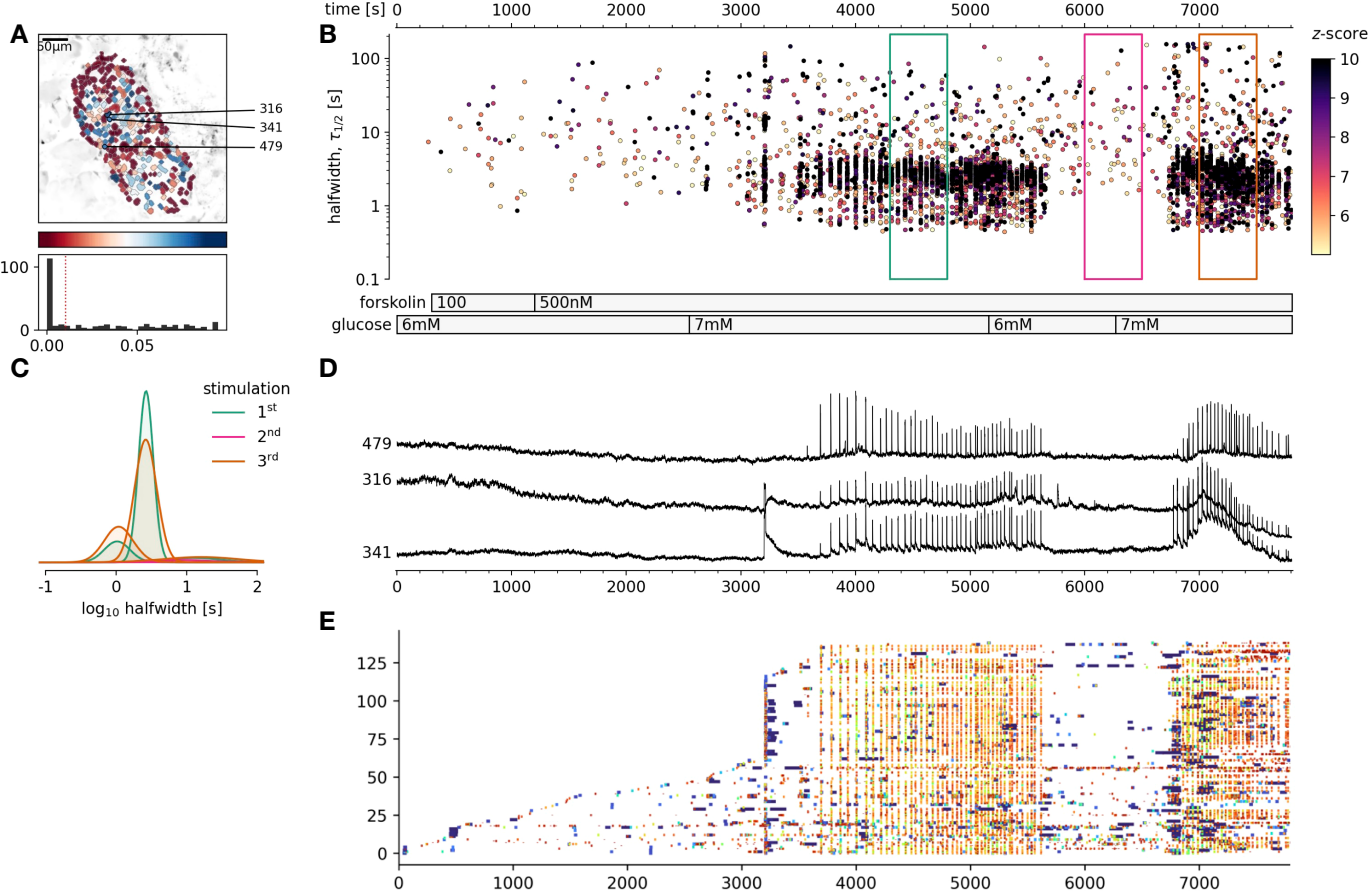
The effect of foskolin stimulation of beta at glucose concentrations around the stimulation threshold. **(A)**, Regions of interest (ROIs) obtained by our segmentation algorithm. The color indicates the number of events identified in the ROI trace, upon a high-pass filtering at 0.2Hz. We discarded ROIs with number of events below the threshold. Indicated are the ROI numbers whose filtered traces correlate best with the average trace for the whole islet. **(B)**, [Ca^2+^]_c_ events’ halfwidth duration through time. Note numerous random [Ca^2+^]_c_ events at 6 mM glucose and cross-correlated activity at 7 mM glucose. Color indicates the statistical significance in terms of z -score as indicated. The treatment protocol is indicated in the bar at the bottom of the pane. **(C)**, Normalized Gaussian fit through the logarithmic distribution of halfwidth. **(D)**, Time courses from ROIs indicated in A, rebinned to 2Hz (recorded at 20Hz). **(A, E)** raster plot of [Ca^2+^]_c_ events sorted by their peak times. Note the progressive recruitment of beta cells at sub threshold glucose concentration and explosive activation, reactivation and cross-correlation with 7 mM glucose. The abscissa is shared for all plots on the right side.

We have demonstrated that cytosolic cAMP concentration could be strongly influenced by extracellular glucose concentration, increasing cAMP concentration over several orders of magnitude. Higher cAMP levels significantly influenced the pattern of [Ca^2+^]_c_ changes in beta cells, particularly influencing the plateau phase activity. The last remaining question was, whether an increase of the cytosolic cAMP in sub-stimulatory glucose concentration would be sufficient to trigger and maintain the coherent activation of beta cells collectives (46). As can be seen in **Figure 4**, forskolin at concentrations sufficient to rescue the adrenalin inhibition, failed to trigger cross-correlated activity in beta cell collectives. Sequent increase of glucose to 7 mM, just above the threshold level triggered a biphasic activation of beta cell collectives (**Figures 4B-D**). Returning glucose concentration back to 6 mM resulted in a complete stop of activity, and switching back to 7 mM glucose, resumed a plateau activity matching the original stimulation measured as frequency and halfwidth duration of [Ca^2+^]_c_ events. It must be however emphasized that 500 nM forskolin increases the frequency of spontaneous [Ca^2+^]_c_ events also at sub threshold glucose concentration (**Figure 4E**). In summary, cAMP concentration is an important cytosolic factor to control the activity of beta cell collectives in situ, however it is not sufficient to activate the cross-correlated activity.

## Discussion

It has been previously shown that glucose stimulation of beta cells can result in the cytosolic accumulation of cAMP (29). This accumulation has been however found to play only a minor role in direct stimulation of insulin release, but exerted a prominent modulation of the process (28). The currently dominant concept regarding pathways regulating the cytosolic level of this potent second messenger molecule would include metabolic, hormonal and neural, but no direct glucose as ligand inputs to beta cells (32). The evidence for a direct glucose sensing that could significantly contribute to the cytosolic production of cAMP has been provided early on (29, 30). This concept, following the so-called receptor hypothesis involves G-protein coupled glucose receptor and has recently received a strong molecular support (38, 39, 47–49). Still, in the last half of a century both concepts regarding glucose-dependent origin of cAMP developed a rather poor intersection. We therefore decided to readdress the role of cAMP in glucose-dependent activation of beta cell collectives and potential complementarity of the concepts mentioned above. To achieve this, we used fresh pancreas slices for advanced imaging and analysis of [Ca^2+^]_c_ events.

Our results suggest, that glucose alone, similarly to forskolin promotes beta cell activity with generation of cAMP in beta cells. The dynamic range of intracellular levels of cAMP are conceivably similarly steeply dependent on stimulatory glucose concentration used. The changes in cytosolic cAMP concentration from any of the possible sources, either directly G-protein mediated, metabolic, hormonal or neural inputs modulate the beta cell activity, possibly leading to changed insulin release. In this study we used the fact that adrenaline exerts its major inhibitory effect on beta cells, likely through α_2_-adrenergic receptors. Such adrenergic receptor activation leads to a decreased AC activity, lowering of cAMP levels and reduced activity of downstream targets, like PKA. In our previous publication we demonstrated that lowered cAMP levels desensitized Ca^2+^ -dependent machinery in late stages of regulated insulin exocytosis through a reduction of the fusion probability of insulin granules (37). In the present study, we upgraded this with the observation that reduced cytosolic cAMP also reduces processes which are upstream to exocytotic events, namely glucose-dependent [Ca^2+^]_c_ events. The events on the plateau phase of the beta cell activity present a stable and reproducible platform to assess the effect of a certain signaling manipulation on [Ca^2+^]_c_ (32, 44, 50). We have previously shown that this plateau activity mainly represents intracellular Ca^2+^ release, in the form of Ca^2+^-induced Ca^2+^ release (CICR), including IP_3_ and ryanodine receptors, which are both sufficient and necessary for this activity (44, 50). Both receptors represent important targets for PKA (51), and their phosphorylation is known to destabilize the receptors and increase the opening probability of the channels and increase CICR (52). We therefore hypothesized that reducing cytosolic cAMP concentration using adrenaline should prevent phosphorylation, stabilize intracellular Ca^2+^ channels, reduce CICR and abolish [Ca^2+^]_c_ events. But why does glucose-independent elevation of cAMP production and PKA activity not lead to destabilization of intracellular Ca^2+^ receptors, followed by an increased insulin release? This can be addressed first classically with a need for increased metabolism, ATP production and initiation of ATP-dependent processes that eventually lead to [Ca^2+^]_c_ and activation of AC (53). Additionally, we need to remind ourselves that specifically in beta cells, ER Ca^2+^ load, and related Ca^2+^ current amplitude during the release from the ER, has a strict glucose dependence (54), and glucose removal results in rapid depletion of the stores (55). Therefore, PKA-dependent destabilization of intracellular Ca^2+^ channels can only be productive when the amplitude of intracellular Ca^2+^ currents is high enough to ignite neighboring channels in the process of CICR (52). Evidence for a version of this concept has been previously provided for INS-1 and mouse beta cells (56).

It is widely accepted that, even at close to threshold glucose concentration, isolated manipulation of the cytosolic cAMP with high concentration of forskolin should not trigger [Ca^2+^]_c_ events on their own (28). Our approach enabled us to have a closer look at this, since it is superior to previous tests, with unprecedented temporal and spatial resolution, combined with automatized detection of both ROIs and events. It is obvious that elevation of cAMP levels at sub stimulatory glucose does not trigger the cross-correlated response in beta cell collectives as stimulatory glucose does, but it does significantly increase the number of spontaneous and non-correlated [Ca^2+^]_c_ events, which are measurable, but likely difficult to pick up as a significant signal within the dynamic range of available insulin hormone release assays.

On the other hand, a drop in the cAMP level due to presence of physiological level of adrenaline could readily and reversibly inhibit beta cell [Ca^2+^]_c_ events on the plateau, stimulated by physiological concentration of glucose. Glucose-dependent processes, which present themselves as cross-correlated activity of beta cell collectives, therefore involve both [Ca^2+^]_c_ concentration needed to trigger activity, as well as a broad range of glucose-concentration-dependent cAMP levels that can provide a whole spectrum of activation and deactivation phenotypes. To support this latter observation, we provide three lines of evidence. Firstly, at a progressively higher stimulatory glucose concentration, a progressively higher adrenaline stimulation of α_2_-adrenergic receptors was required to inhibit the beta cell activity. More quantitatively, doubling of stimulatory glucose concentration stimulated beta cell collective activity so high that two orders of magnitude higher concentration of adrenaline were required to inhibit [Ca^2+^]_c_ events. Secondly, subsequent application of a higher glucose concentration could override the inhibitory effect of adrenaline obtained a lower glucose concentration, and this activity could only be inhibited with a non-linearly higher adrenaline concentration. And thirdly, specific elevation of cAMP level with forskolin could override the inhibitory adrenaline effect in a similar way to glucose.

The non-linear relationship between the glucose-concentration and beta cell activity may have significant repercussions. Firstly, regarding the interpretations of the results of the experiments obtained at acute supraphysiological glucose concentrations, where cAMP levels may be orders of magnitude above the physiological levels and with the downstream effectors maximally activated. This scenario is realistic in culturing beta cell, where high glucose levels in culture media are common. And secondly, it could provide novel insights regarding the efficiency of the sympathoadrenal system in the pathogenesis of hyperglycemia and diabetes mellitus, as it has been shown early on that the catecholamines are elevated in diabetes. Long-term hyperglycemia during the prediabetic phases due to progressive insulin resistance could wind up cytosolic cAMP levels and challenge the efficiency of the sympathoadrenal system. In theory, in diabetic context the influence of sympathoadrenal system can either be increased or decreased. It has been previously demonstrated that in diabetic rats α_2_A-adrenergic receptors get upregulated (23). However, such overexpression could on the long run result in downregulation of downstream proteins involved in cAMP production, and lowering cytosolic cAMP levels and inducing beta cells stress, followed by a reduced function. This would also be one possible explanation why in type 2 diabetics, the sensitivity to GLP-1 is severely impaired (57).

In some previous studies, adrenaline in physiological concentrations has been applied to study insulin release inhibition in beta cells. Rat isolated islet were susceptible to 0.1 nM adrenaline, whereas almost complete insulin secretion inhibition was achieved with 100 nM adrenaline, when isolated islets were stimulated with 15 mM glucose concentration (13). Similarly, adrenaline concentration dependency on insulin secretion inhibition in mice was also previously described. Adrenaline at 0.1 nM in the presence of 15 mM glucose had no effect. Partial inhibition was observed at 1 nM adrenaline and it was almost complete at 1 µM adrenaline (14). These results are in good agreement with our data. When beta cells were stimulated with similar glucose concentration (16 mM), adrenaline in the same concentration range was required to inhibit [Ca^2+^]_c_ events. Also glucose dependence of adrenaline inhibition has been previously shown using electrical activity measurements (58). In the present study we confirmed these observations at more physiological and *in situ* conditions with improved spatial and temporal resolution.

It is worth mentioning that PKA and intracellular Ca^2+^ release may not be the exclusive target of cAMP, as exchange protein directly activated by cAMP (EPAC2) (59). In addition, adrenaline has been shown to attenuate insulin release *via* coupling of α2A-adrenoceptor to cAMP/TRPM2 signaling transient receptor potential melastatin 2 (TRPM2), a type of nonselective cation channel (NSCC), which affects membrane potential and contributes to inhibition of beta cells (15). In beta cells in tissue slices we were not able to reproduce PKA-dependent changes in the opening probability of L-type voltage-activated channels, although we did not explore the whole concentration range (37).

It has been reported that [Ca^2+^]_c_ events in beta cells are well coordinated with cAMP oscillations (35, 36) and that these oscillations together are crucial for insulin release. When [Ca^2+^]_c_ in beta cells is elevated, metabolism in the cell is a potent trigger for cAMP production (35). Moreover, elevation in Ca^2+^ levels is sufficient to trigger rise of cAMP levels in beta cell (60). Interestingly, [cAMP]_c_ response can be augmented by Ca^2+^, however at the same time appears [Ca^2+^]_c_ oscillations are not essential for glucose-induced oscillations of [cAMP]_c_ (36), since in intact islets [cAMP]_c_ oscillations were preserved (but suppressed) after glucose-stimulated Ca^2+^ influx was prevented or Ca^2+^ was removed from the extracellular space (35). New findings, giving Ca^2+^ influx a minor role and suggesting intracellular Ca^2+^ stores and receptors play a key role in shaping glucose-dependent [Ca^2+^]_c_ responses (44) could weaken the interpretation discussed above.

Since islet containing pancreas tissue slices that were subjected to Ca^2+^ imaging were chosen randomly, it is only logical to assume the observed variability could arise from regional differences in density of sympathetic innervation, since it was reported that head and neck regions contain higher levels of NA in the ganglia (61). Variability could also be due to different density in α- and β-adrenergic receptors expressed on beta cells (2). NA and adrenaline were shown to have also stimulatory effects on insulin secretion, through direct and indirect actions. Firstly, glucagon released by alpha cells activated by the sympathoadrenal system (62), can stimulate insulin release from beta cells (2). Secondly, it is considered that sympathoadrenal system activates β_2_-adrenoceptors on beta cells directly to stimulate insulin secretion (63). The net effect of physiological actions of catecholamines and NA released by postganglionic sympathetic nerve fibers on insulin secretion is dependent on density of α- and β-adrenergic receptors expressed on beta cells. For this reason the net effect of sympathoadrenal system on insulin secretion from pancreatic beta cells is likely a mélange of inhibitory and stimulatory effects on insulin secretion (2). We could readily observe the long-term stimulatory effects of adrenaline on alpha cell function, however under our experimental conditions, only a mild and transient positive effect of low concentration of adrenaline which resulted in an elevated bursting frequency soon after the addition of low adrenalin concentration. Such transient boost of insulin secretion at the beginning of the stress condition could help to provide faster availability of nutrients to critical tissues.

To conclude, glucose stimulate both intracellular Ca^2+^ release, and as suggested by our adrenaline inhibition data, a broad range of activities that could similarly as forskolin produce changes in the cytosolic concentration of cAMP in beta cells. Consequently, cAMP levels could exert a wide range shaping of activation and deactivation patterns of these cells *in situ* in fresh pancreas tissue slices.

## Data availability statement

The original contributions presented in the study are included in the article/supplementary materials. Further inquiries can be directed to the corresponding author.

## Ethics statement

The animal study was reviewed and approved by The Ministry of Education, Science and Research, Republic of Austria (Licence No: 2020-0.488.800), Administration of the Republic of Slovenia for Food Safety, Veterinary Sector and Plant Protection (Licence No: U34401-12/2015/3).

## Author contributions

Conceptualization, NS and MSR. Methodology, NS, SP, JP, LKB, JK, MSK, SS, AS, and DK. Writing-original draft preparation, NS, and MSR. Writing-review and editing, NS, SP, JP, LKB, JK, MSK, SS, AS, DK, and MSR. Funding acquisition, AS and MSR. All authors have read and agreed to the published version of the manuscript.

## Funding

MR receives grants by the Austrian Science Fund/Fonds zur Förderung der Wissenschaftlichen Forschung (bilateral grants I3562-B27 and I4319-B30), and from NIH (R01DK127236). MR and AS further received financial support from the Slovenian Research Agency (research core funding programs P3-0396 and I0-0029, as well as projects N3-0048, N3-0133 and J3-9289).

## Conflict of interest

The authors declare that the research was conducted in the absence of any commercial or financial relationships that could be construed as a potential conflict of interest.

## Publisher’s note

All claims expressed in this article are solely those of the authors and do not necessarily represent those of their affiliated organizations, or those of the publisher, the editors and the reviewers. Any product that may be evaluated in this article, or claim that may be made by its manufacturer, is not guaranteed or endorsed by the publisher.
